# The Pharmaceutical Industry in 2023: An Analysis of FDA Drug Approvals from the Perspective of Molecules

**DOI:** 10.3390/molecules29030585

**Published:** 2024-01-25

**Authors:** Beatriz G. de la Torre, Fernando Albericio

**Affiliations:** 1 Kwazulu-Natal Research Innovation and Sequencing Platform (KRISP), College of Health Sciences, University of KwaZulu-Natal, Durban 4001, South Africa; 2 School of Chemistry and Physics, University of KwaZulu-Natal, Durban 4001, South Africa; 3 Department of Organic Chemistry, University of Barcelona, 08028 Barcelona, Spain

**Keywords:** antibodies, biologics, chemical entities, fluorine-based drugs, imaging, natural products, new chemical entities, oligonucleotides, peptides, TIDES

## Abstract

With the COVID-19 pandemic behind us, the U.S. Food and Drug Administration (FDA) has approved 55 new drugs in 2023, a figure consistent with the number authorized in the last five years (53 per year on average). Thus, 2023 marks the second-best yearly FDA harvest after 2018 (59 approvals) in all the series. Monoclonal antibodies (mAbs) continue to be the class of drugs with the most approvals, with an exceptional 12, a number that makes it the most outstanding year for this class. As in 2022, five proteins/enzymes have been approved in 2023. However, no antibody–drug conjugates (ADCs) have been released onto the market. With respect to TIDES (peptides and oligonucleotides), 2023 has proved a spectacular year, with a total of nine approvals, corresponding to five peptides and four oligonucleotides. Natural products continue to be the best source of inspiration for drug development, with 10 new products on the market. Three drugs in this year’s harvest are pegylated, which may indicate the return of pegylation as a method to increase the half-lives of drugs after the withdrawal of peginesatide from the market in 2013. Following the trends in recent years, two bispecific drugs have been authorized in 2023. As in the preceding years, fluorine and/or N-aromatic heterocycles are present in most of the drugs. Herein, the 55 new drugs approved by the FDA in 2023 are analyzed exclusively on the basis of their chemical structure. They are classified as the following: biologics (antibodies, proteins/enzymes); TIDES (peptide and oligonucleotides); combined drugs; pegylated drugs; natural products; nitrogen aromatic heterocycles; fluorine-containing molecules; and other small molecules.

## 1. Analysis

A year ago, we embarked on a new “normality” after the COVID-19 pandemic. Fortunately, this year (2023), we can confirm a scenario in which the disease is being treated in many countries like other diseases such as flu, which also causes a large number of deaths worldwide every year. An important outcome of the pandemic has been the establishment of mRNA technology for the development of vaccines for other diseases, such as cancer. This progress, accompanied by the incipient rise of oligonucleotides as new chemical entity (NCE) drugs, may indicate that the pharmaceutical industry is entering a new era with respect to nucleotides.

In 2023, 55 new drugs have been approved by the U.S. Food and Drug Administration (FDA) [1]. This is the second-highest number, after 59 approvals in 2018. This year’s figure responds to one of the questions raised last year from when we analyzed the 37 drugs authorized by the FDA in 2022 [2]. This relatively low number in comparison with previous years [59 drugs accepted in 2018, 48 in 2019, 53 in 2020, and 50 in 2021] could be considered a temporary glitch rather than a consequence of the COVID-19 pandemic. Indeed, the pharmaceutical industry has been reinforced by the pandemic, as shown by the large number of drugs approved and those in the pipeline.

**Figure 1:**
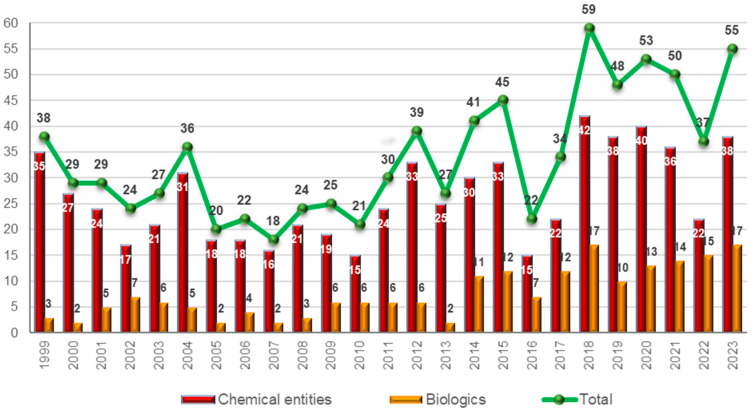
Drugs (new chemical entities and biologics) approved by the FDA in the last 25 years. Adapted with permission from ref. [2]. Copyright 2023, copyright MDPI [1,2,3].

The 55 drugs approved this year are divided between 17 biologics (17, 10, 13, 14, and 15 in 2018, 2019, 2020, 2021, and 2022, respectively), 9 TIDES (5 peptides and 4 oligonucleotides in 2023 against 1 and 3 in 2018; 3 and 2 in 2019 and 2020; 8 and 2 in 2021; and 4 and 1 in 2022), and 29 so-called small molecules, in total 38 NCEs (TIDES and small molecules) (Figure 1) [1,2,3]. In the distribution between biologics and NCEs, 2023 can be considered a standard year, with 17 biologics vs. an average of 14 in the last five years and 38 NCEs vs. an average of 36 in the same period. In 2023, biologics accounted for 30% of all drugs, while in the last five years, this figure was approximately 28% (69 biologics out of a total of 247 drugs) [2].

In addition, the Center for Biologics Evaluation and Research (CBER) has added 22 new Biologics License Application approvals in 2023, almost double those registered in 2021 (13) and 2022 (12) [4], including several vaccines for the respiratory syncytial virus, the chikungunya virus, and meningococcal groups A, B, C, W, and Y, among others.

Herein, the 55 new drugs approved by the FDA in 2023 are analyzed exclusively based on their chemical structure. To keep a good flow of the manuscript, we have kept the number of references at a minimum. Further information can be obtained in ref. [1].

## 2. Discussion

Table 1 shows the 17 biologics approved in 2023, of which 12 are monoclonal antibodies (mAbs), which represents the best year for this kind of drugs, and 5 are enzymes/proteins (Table 1).

Regarding biologics, and following the trend of recent years, 2023 has seen the approval of two bispecific mAbs. These drugs can simultaneously bind to two distinct epitopes on one antigen or to two different antigens. In this regard, authorization was given to glofitamab-gxbm (Columvi^TM^), a bispecific CD20-directed CD3 T-cell engager, and elranatamab (Elrexfio^TM^), which targets BCMA (a protein on myeloma cells) and CD3 (a protein on the immune system’s T-cells). In 2022, three of the biologics approved showed bispecificity [teclistamab-cqyv (Tecvayli^TM^), faricimab-svoa (Vabysmo^TM^), and tebentafusp-tebn (Kimmtrak^TM^)], while only one was approved in 2021 [amivantamab-vmjw (Rybrevant^TM^)], 2018 [emicizumab (Hemlibra^TM^)], and 2014 [blinatumomab (Blincyto^TM^)].

Last year, we highlighted the controversy around aducanumab-avwa (Aduhelm^TM^), which was approved in 2021 for the treatment of Alzheimer’s disease and whose effectiveness was under discussion. In 2023, lecanemab (Leqembi^TM^) has been approved for the same target. This drug was developed by Biogen—the same company that manufactured aducanumab-avwa—in association with Eisai.

mAbs continue to be the most approved drug class, accounting for 22% (12 vs. 55) of all the drugs accepted by the FDA this year. In 2022, these drugs accounted for 24% (9 vs. 37). As in former years, cancer is the first target for mAbs. However, following the trend of previous years, mAbs have been approved for other targets such as Alzheimer’s disease, psoriasis, respiratory syncytial virus, ulcerative colitis, and myasthenia gravis, thus enlarging the application of these kinds of challenging drugs.

The streak has been broken in 2023 regarding the approval of antibody–drug conjugates (ADCs) as none have been authorized, a situation that contrasts with previous years, in which up to 14 such drugs were accepted by the FDA.

This year, three enzyme replacement therapies have received the green light. Velmanase (Lamzede^TM^) is the first such therapy approved for the treatment of non-central nervous system manifestations of α-mannosidosis. Pegunigalsidase alfa (Elfabrio^TM^), which is a pegylated, crosslinked, chemically modified recombinant human α-galactosidase A enzyme, is indicated for the treatment of Fabry disease. Finally, cipaglucosidase alfa-atga (Pombiliti^TM^) provides an exogenous source of α-glucosidase for the treatment of Pompe disease.

TIDES (oligonucleotides and peptides), although they belong to the class of chemical entities, often show structural complexity similar to that of biologics. TIDES are chemically synthesized, and their characterization and the profile of impurities allowed by regulatory agencies are similar to what is required for small molecules, thereby making their introduction into the market a real challenge. In 2023, nine TIDES (five peptides and four oligonucleotides) have been approved, which account for 16% (9 vs. 55) of all the drugs authorized this year—almost the same proportion as in 2022 (15%) (5 vs. 37)—thus reinforcing the importance of these kinds of drugs. As is our custom in these yearly reports, we consider peptidomimetics with a high presence of amino acids or peptide bonds to be peptides.

The last few years have witnessed a double trend in the peptides approved by the FDA and other regulatory agencies. On the one hand, authorization has been given to large linear peptides with more than 30 amino acids, whose best exponent was the approval of tirzepatide (Mounjaro^TM^) in 2022 for the treatment of type 2 diabetes and obesity. On the other hand, cyclic peptides of medium size often contain non-proteinogenic amino acids. In both cases, pending units of fatty acids or/and polyethyleneglycol (PEG) structures are present to increase the drugs’ half-lives. In 2023, three cyclic peptides with these characteristics have been approved.

**Figure 2:**
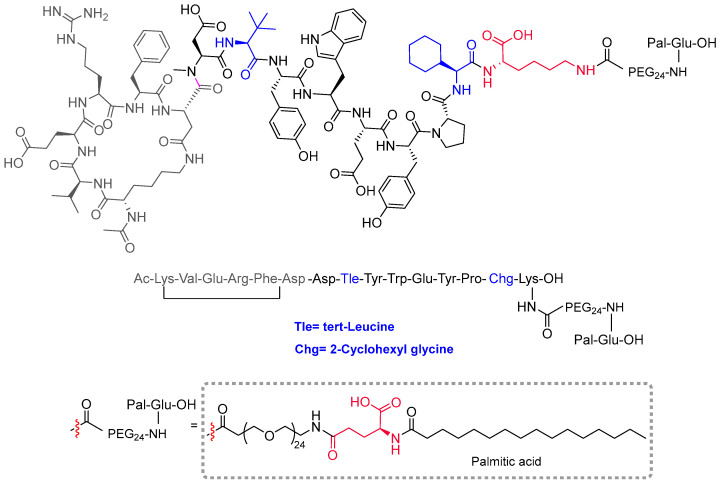
Structure of zilucoplan (blue, non-proteinogenic amino acids; red, γ-amide bond).

Zilucoplan (Zilbrysq^TM^) (Figure 2) is recommended for the treatment of myasthenia gravis in adults who are anti-acetylcholine-receptor-antibody-positive. Zilucoplan binds to protein complement component 5 (C5) and inhibits its cleavage into two fragments. It is a side-chain-to-side-chain homodetic cyclic peptide with a main backbone of 15 amino acids, all of them in the L-configuration. The cyclic structure is at the N-terminal part between the side chains of the Lys and Asp residues. A Lys residue situated at the C-terminal part bears a side chain containing a large PEG and a fatty structure (palmitic acid) linked by a γ-Glu residue. The combination of fatty acids linked by a γ-Glu residue to extend the half-life of the molecule in vivo is present in other peptides, including liraglutide, semaglutide, and tirzepatide, all of which were approved in recent years. The backbone of zilucoplan contains several non-proteinogenic amino acids such as NMe-Asp, tert-butylglycine or tert-leucine, aza-tryptophan, and cyclohexylglycine. The N-terminus is acylated and the C-terminus is a free carboxylic group.

**Figure 3:**
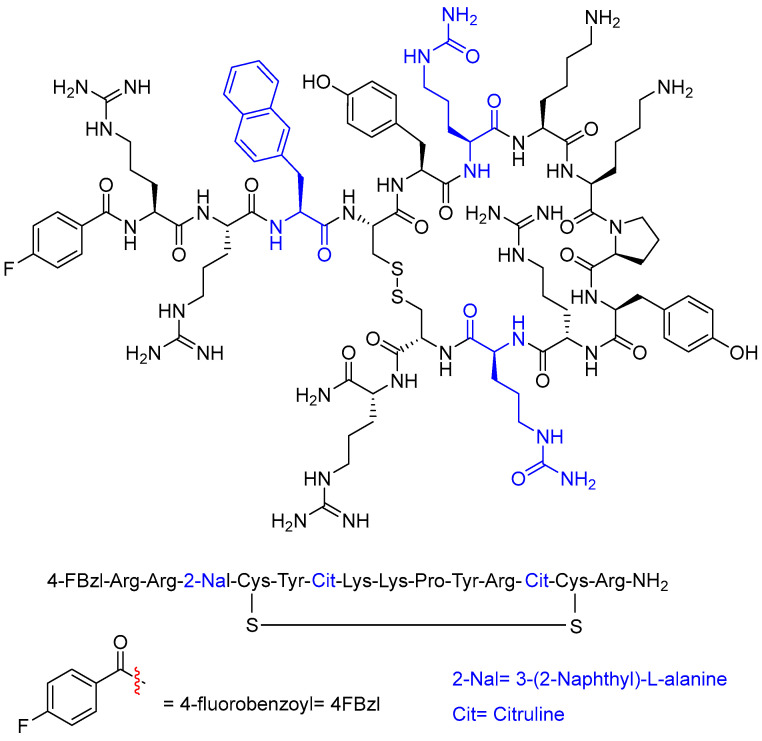
Structure of motixafortide.

Motixafortide (Aphexda^TM^) (Figure 3) has been approved for the treatment of multiple myeloma. It is a hematopoietic stem cell mobilizer and a CXCR4 antagonist. Motixafortide is a 14-amino-acid cyclic heterodetic peptide formed by a disulfide bridge between two Cys residues. It is a cationic peptide with four Arg and two Lys residues. In addition, it contains two Cit residues and one naphthylalanine. The N-terminus is in the form of p-fluorobenzoyl, and the C-terminus is amidated.

**Figure 4:**
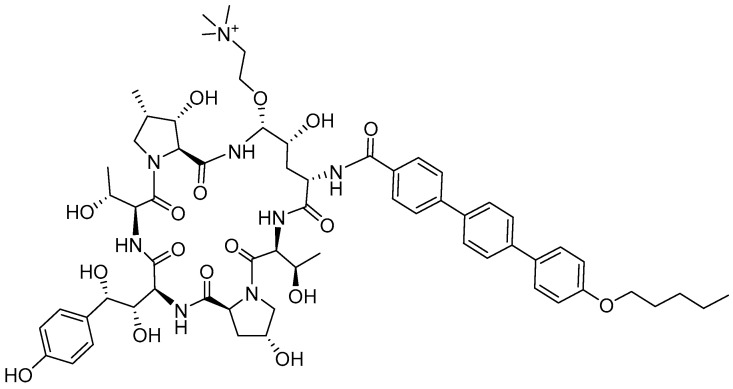
Structure of rezzayo.

Rezzayo (Rezafungin^TM^) (Figure 4) is an antifungal drug of the echinocandin family indicated for the treatment of candidemia and invasive candidiasis. It is a semi-synthetic hexapeptide side chain tailing a homodetic cyclic peptide. It is a highly hydroxylated peptide, which, in addition to having two Thr residues, contains hydroxyproline (2), hydroxyornitine, and hydroxyhomotyrosine residues. The side chain of the Orn derivative contains a choline moiety. The cycle is formed by the C-terminus of one of the hydroxyprolines and the δ-amino of the Orn. The N-amino is in the form of pentoxyphenylphenylbenzamide.

**Figure 5:**
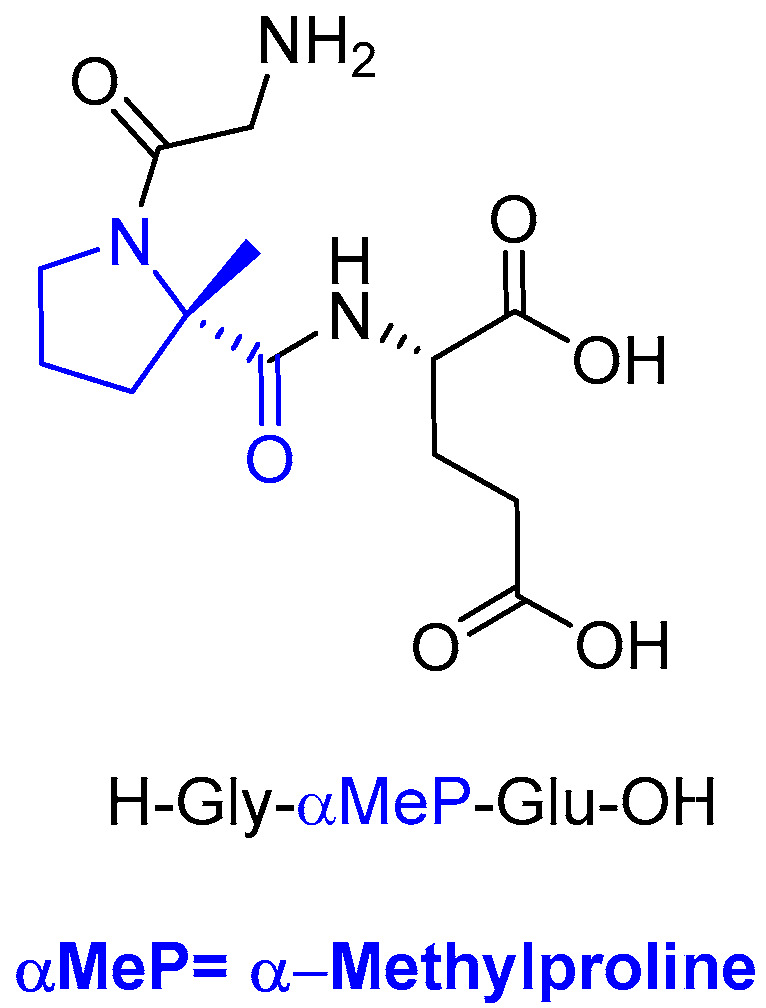
Structure of trofinetide.

Trofinetide (Daybue^TM^) (Figure 5) is an orally available tripeptide containing two proteinogenic amino acids, Gly and Glu, and a residue of α-methylproline, and it is used for the treatment of Rett syndrome. It is the methylated analog of the N-terminal tripeptide (Gly-Pro-Glu) of the insulin-like growth factor 1 protein.

**Figure 6:**
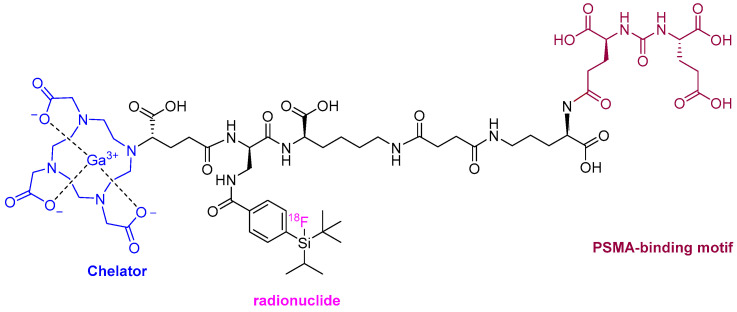
Structure of flotufolastat F-18.

Following last year’s trends, a radiopharmaceutical agent for use in positron emission tomography (PET) imaging for prostate cancer has been approved this year. Flotufolastat F-18 (Posluma^TM^) (Figure 6) is the fourth prostate-specific membrane antigen (PSMA)-targeted PET imaging drug to be authorized after lutetium (177Lu) vipivotide tetraxetan (Pluvicto^TM^) in 2022, piflufolastat F-18 (Pylarify^TM^) in 2021, and Ga-68 PSMA-1 in 2020.

The chemical structure of flotufolastat F-18 differs from the previous PSMA-targeted drugs, which were based on the urea of Glu and Lys. In the case of this year’s drug, the urea is formed from the α-amino of two Glu residues that are linked to a DOTAGA complex with non-radioactive Ga and radioactive F-18 covalently bound to a silicon moiety, through a linker formed of four residues: D-Orn, D-Lys, succinic acid, and D-diaminopropionic acid (Dap).

With the FDA approval of four oligonucleotide-based drugs this year, there are now 19 such drugs available, 16 of which have emerged since 2016, the year that marked a new era for oligos as drugs.

**Figure 7:**
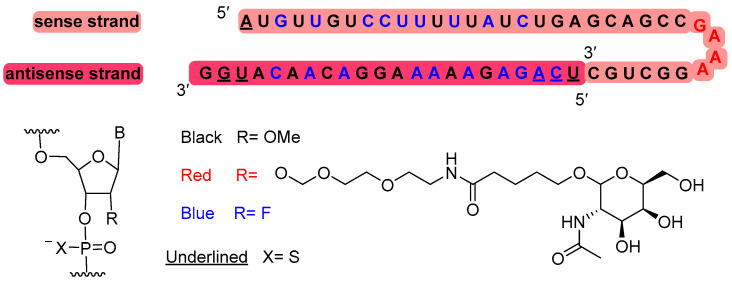
Structure of nedosiran.

Nedosiran (Rivfloza^TM^) (Figure 7) is recommended for the treatment of primary hyperoxaluria. It is a double-stranded small interfering RNA (siRNA) containing four units of N-acetyl-D-galactosamine (GalNAc) residues, which are responsible for targeting lactate dehydrogenase A (LDHA) in hepatocytes. Nedosiran is formed of 36 and 22 ribonucleotides in the sense and antisense strands, respectively. It has a total of six thiophosphate linkages. In addition to the GalNAc residues, nedosiran contains 19 2′-F-ribonucleotide to improve the stability of the double strand. The remaining ribonucleotides are 2′-methoxy.

**Figure 8:**
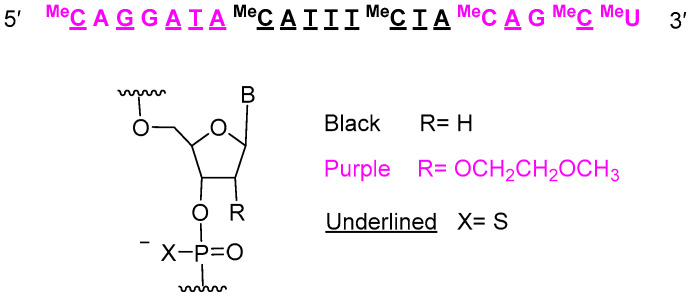
Structure of tofersen.

The remaining oligonucleotides approved in 2023 are single-strain antisense oligonucleotides. Tofersen (Qalsody^TM^) (Figure 8) is used for the treatment of amyotrophic lateral sclerosis (ALS). It contains 15 thiophosphate linkages and is formed of 20 nucleotides: 10 metoxyethoxy and 10 deoxy.

**Figure 9:**
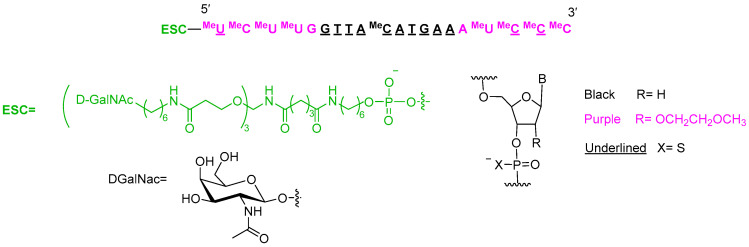
Structure of eplontersen.

Eplontersen (Wainua^TM^) (Figure 9) is indicated for the treatment of transthyretin-mediated amyloidosis. It contains 11 thiophosphate linkages; is formed, again, of 20 nucleotides (10 metoxyethoxy and 10 deoxy); and ends with a trivalent N-acetylgalactosamine (GalNAc) [Enhanced Stabilization Chemistry (ESC)], which mediates the binding and internalization of the drug by hepatocytes. This system is present in many oligonucleotides that have recently reached the market.

**Figure 10:**
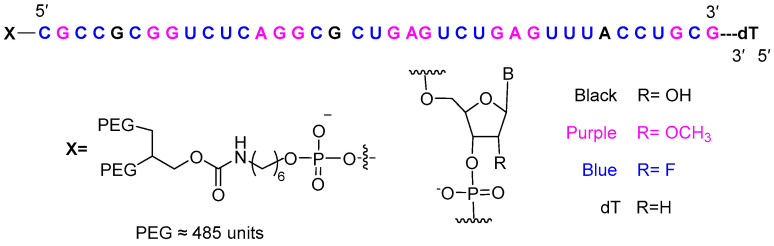
Structure of avacincaptad pegol.

Finally, avacincaptad pegol (Izervay^TM^) (Figure 10) is recommended for the treatment of age-related macular degeneration. Containing 39 nucleotides, this modified aptamer incorporates an inverted thymidine nucleotide to cap the 3′ end, 2′-fluorine (21), and O-methyl (14)-modified nucleotides, together with hydroxy nucelotides (3). It is terminated in two PEG chains, each with approximately 485 units. These modifications extend the half-life of the drug in vivo. Avacincaptad pegol functions as a chemical antibody against complement C5 and inhibits the cleavage of complement C5 into its two fragments—a similar mode of action to that of the peptide zilucoplan, which was also approved this year.

**Figure 11:**
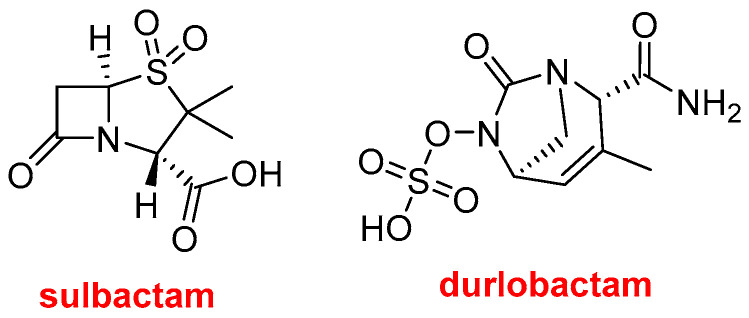
Structures of sulbactam and durlobactam contained in Xacduro^TM^.

This year, the FDA has also authorized three drugs that contain more than one active pharmaceutical ingredient (API). Xacduro^TM^ (Figure 11), which contains sulbactam and durlobactam, is used for the treatment of bacterial pneumonia caused by the Acinetobacter baumannii–calcoaceticus complex. Sulbactam is a β-lactam antibacterial and β-lactamase inhibitor, and durlobactam is a β-lactamase inhibitor.

Sulbactam was first approved in 1986 and is already used in combination with ampicillin (Unasyn^TM^) and cefoperazone (Sulperazon^TM^).

**Figure 12:**
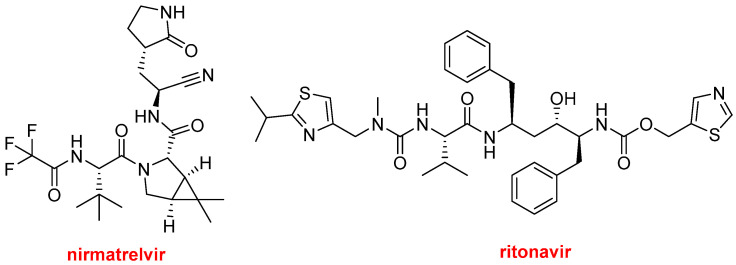
Structures of nirmatrelvir and ritonavir contained in Paxlovid^TM^.

Paxlovid^TM^ (Figure 12), which contains nirmatrelvir and ritonavir, has been officially approved this year to treat mild to moderate COVID-19 to avoid progression to severe disease. This drug already received emergency authorization in 2021 and 2022 in the USA and other countries, respectively, for this purpose. There has been some controversy regarding its efficacy and the COVID-19 variants most likely to be susceptible to this treatment.

Nirmatrelvir is a protease inhibitor used as an antiviral drug. Ritonavir (Norvir^TM^), approved in 1996, is also a protease inhibitor that is used as an antiretroviral in combination with other drugs to treat HIV/AIDS and hepatitis C, among others. In 2014 and 2015, ritonavir, ombitasvir, and paritaprevir, in combination with dasabuvir or ribavirin, were approved to treat the hepatitis C virus.

In Paxlovid^TM^, ritonavir slows the metabolism of nirmatrelvir via cytochrome enzyme inhibition and therefore reinforces the role of nirmatrelvir, which is the main drug.

**Figure 13:**
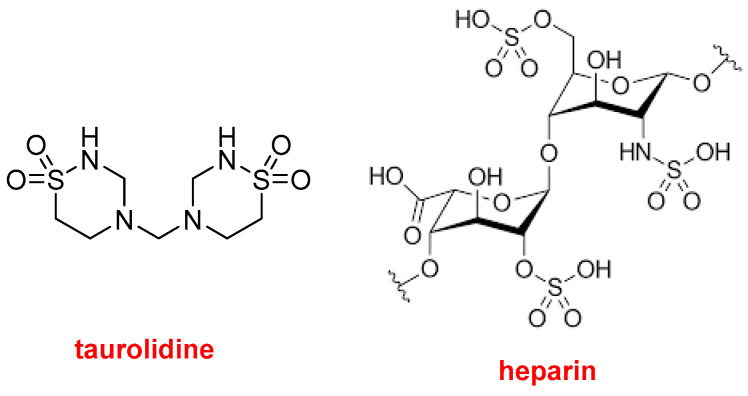
Structures of taurolidine and heparin contained in Defencath^TM^.

Defencath^TM^ (Figure 13) contains taurolidine, a thiadiazinane antimicrobial compound, and the glycosaminoglycan polymer heparin, an anticoagulant. It is used as a catheter lock solution for central venous catheter instillation. Taurolidine is derived from taurine, which is an endogenous amino acid.

A total of 22 combination drugs have been approved by the FDA between 2016 and 2023. This figure illustrates the relevance of this strategy for drug discovery, which often takes advantage of “old” drugs already approved by regulatory agencies.

Natural products are one of the most important sources of inspiration for developing new drugs. In addition to the biologics, TIDES, sulbactam, taurolidine, and heparin cited above, six more drugs approved this year by the FDA found their roots in the natural product universe.

**Figure 14:**
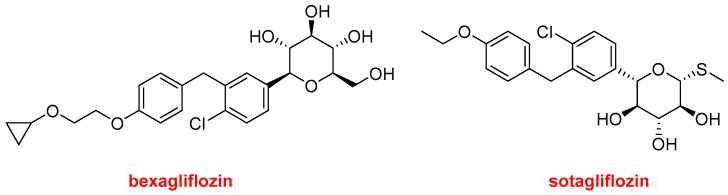
Structures of bexagliflozin and sotagliflozin.

Bexagliflozin (Brenzavvy^TM^) and sotagliflozin (Inpefa^TM^) (Figure 14) are two related inhibitors of sodium–glucose cotransporter 2 (SGLT2).

The former has been authorized for improving glycemic control in adults with type 2 diabetes in combination with exercise and a corresponding diet. In contrast, sotagliflozin has been approved only this year by the FDA for reducing the risk of death due to heart failure. However, in Europe, sotagliflozin (Zynquista^TM^) was approved in 2019 for the treatment of type 1 diabetes, but it was withdrawn in 2022. The FDA has refused its approval in combination with insulin for the treatment of type 1 diabetes.

**Figure 15:**
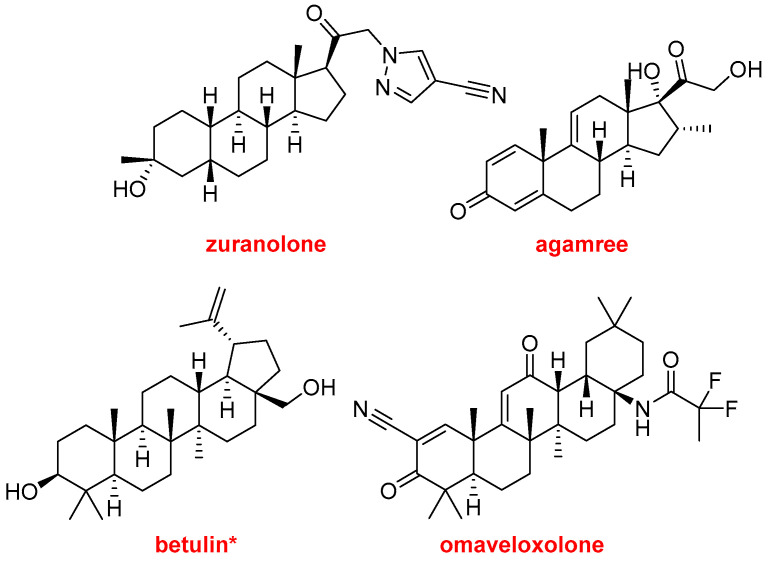
Structures of zuranolone, agamree, betulin*—the main component of birch triterpenes—and omaveloxolone.

Zuranolone (Zurzuvae^TM^) and agamree (Vamorolone^TM^) (Figure 15) belong to the steroid family and have been approved for the treatment of postpartum depression and Duchenne muscular dystrophy, respectively.

Birch triterpenes (Filsuvez^TM^) (Figure 14) could be considered typical natural product drugs because they are botanical drug substances composed of a mixture of pentacyclic triterpenes: botulin (72–88%), lupeol (2.4–5.7%), betulinic acid (2.6–4.2%), erythrodiol (0.5–1.2%), and oleanolic acid (0.3–0.8%). They are used in a topical gel indicated for the treatment of partial-thickness wounds with junctional and dystrophic epidermolysis bullosa (JEB and DEB). This is the first treatment approved for wounds associated with JEB, which is considered a rare disease.

Omaveloxolone (Skyclarys^TM^) (Figure 14) is recommended for the treatment of Friedrich’s ataxia. The FDA has granted Orphan Drug, Fast Track, Priority Review, and Rare Pediatric Disease designations to omaveloxolone [5].

Steroids and triterpenes are the most important class of small-molecule-based drugs, as is reflected by their approval year after year.

Every year, we highlight the importance of F in the drug discovery arena, and 2023 has been no exception. Thus, in addition to two peptides (motixafortide and flotufolastat F-18); two oligonucleotides (nedosiran and avacincaptad pegol); nirmatrelvir as part of Paxlovid^TM^; and omaveloxolone, eight more APIs containing F have been authorized. Thus, 25% (14 vs. 55) of all approved drugs contain F, and if biologics are omitted from the calculation, this figure increases to 37% (14 vs. 38).

Possibly the simplest but also one of the most interesting F-containing drugs is perfluorhexyloctane (Miebo^TM^) (Figure 15). This semi-fluorinated alkane is used to treat dry eye disease. The presence of F makes an alkane druggable.

Repotrectinib (Augtyro^TM^) (Figure 15) is the only macrocycle approved this year for the treatment of non-small-cell lung cancer. It is an inhibitor of proto-oncogene tyrosine-protein kinase ROS1 and of the tropomyosin receptor tyrosine kinases (TRKs) TRKA, TRKB, and TRKC.

Lotilaner (Xdemvy^TM^) (Figure 15) is an antiparasitic drug used to treat the inflammation of the eyelid caused by infestation by the tiny mites Demodex. It contains two CF_3_ groups in an extended polyheterocyclic structure, which is a common feature of other F-containing drugs.

**Figure 16:**
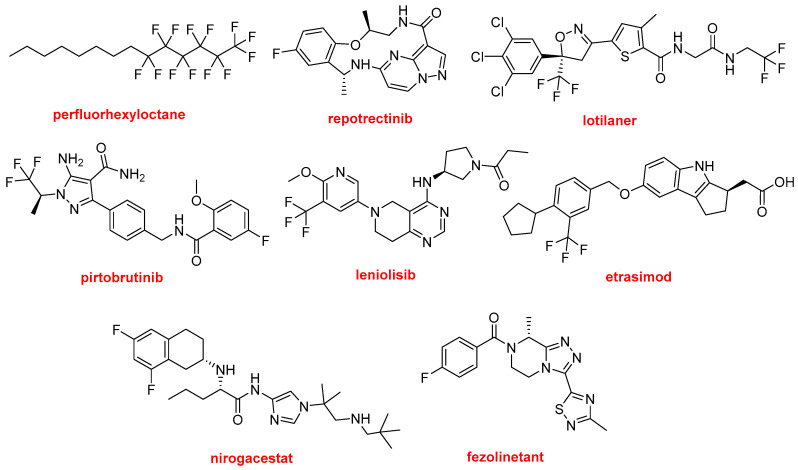
Structures of perfluorhexyloctane, repotrectinib, lotilaner, pirtobrutinib, leniolisib, etrasimod, nirogacestat, and fezolinetant, all F-containing APIs.

There are three drugs approved containing a CF_3_ moiety (Figure 16). Pirtobrutinib (Jaypirca^TM^) is indicated to treat mantle cell lymphoma. It is a Bruton’s tyrosine kinase (BTK) inhibitor, acting on B-cell lymphocyte proliferation and survival. In addition to the CF_3_ group, pirtobrutinib contains a F-phenyl moiety. Leniolisib (Joenja^TM^) is another kinase inhibitor used to treat activated phosphoinositide 3-kinase delta syndrome. Etrasimod (Velsipity^TM^) is an immune modulator recommended for the treatment of ulcerative colitis. Nirogacestat (Ogsiveo^TM^), which contains a difluorophenyl moiety, is a selective γ-secretase inhibitor used to treat desmoid tumors. Finally, fezolinetant (Veozah^TM^) is indicated for the treatment of the vasomotor symptoms (hot flushes) caused by menopause.

**Figure 17:**
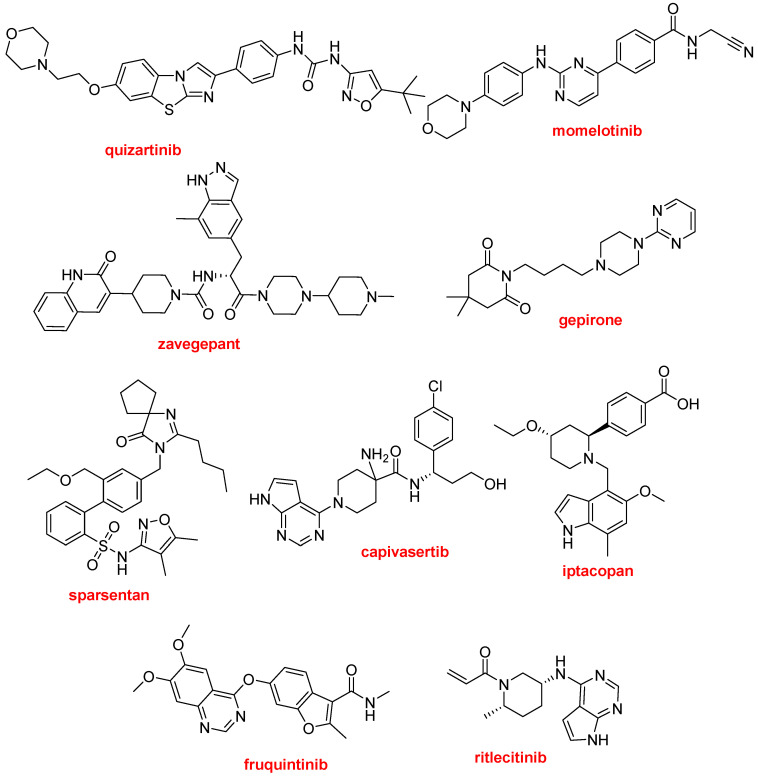
Structures of quizartinib, momelotinib, zavegepant, gepirone, sparsentan, capivasertib, iptacopan, fruquintinib, and ritlecitinib, extended polyheterocyclics with N as major heteroatom.

Six of the seven F-containing drugs, as well as the nirmatrelvir and ritonavir present in Paxlovid^TM^, could be considered extended polyheterocyclics with N as a major heteroatom. In addition to these nine APIs, there are nine more from the same group, which represents 33% (18 vs. 55) of all approved drugs (Figure 17).

Quizartinib (Vanflyta^TM^), a clear example of this kind of molecule, is indicated for the treatment of acute myeloid leukemia. It is a tyrosine kinase inhibitor, targeting the proto-oncogene FLT3 or CD135.

Momelotinib (Ojjaara^TM^) is a Janus kinase inhibitor used for the treatment of myelofibrosis. Like the previous drug, it has a morpholine moiety at one end.

Zavegepant (Zavzpret^TM^) is a calcitonin gene-related peptide receptor antagonist indicated for the treatment of migraines.

Gepirone (Exxua^TM^) is a clear example of the difficulty of marketing a new drug. It was first synthesized in 1986 and was rejected three times by the FDA before its approval in 2023 for the treatment of major depressive disorder. Gepirone is a partial agonist of the serotonin 5-HT1A receptor, and one of its metabolites is an α2-adrenergic receptor antagonist.

Sparsentan (Filspari^TM^), the only sulfonamide approved this year, is indicated for the treatment of primary immunoglobulin A nephropathy. Sparsentan is an endothelin and angiotensin II receptor antagonist.

Capivasertib (Truqap^TM^) is used in combination with fulvestrant (Faslodex^TM^) for the treatment of patients with hormone-receptor-positive, human-epidermal-growth-factor-receptor-2-negative breast cancer with metastatic disease.

Iptacopan (Fabhalta^TM^), a complement factor B inhibitor, is approved for paroxysmal nocturnal hemoglobinuria.

Fruquintinib (Fruzaqla^TM^) is a kinase inhibitor indicated for the treatment of metastatic colorectal cancer.

Ritlecitinib (Litfulo^TM^) is a kinase inhibitor, acting on Janus kinase 3 and tyrosine kinase, used for the treatment of severe hair loss (alopecia areata).

Palovarotene (Sohonos^TM^) and daprodustat (Duvroq^TM^) (Figure 17) also contain at least one N-heterocycle. The former is indicated to reduce the volume of new heterotopic ossification in adults and pediatric patients with fibrodysplasia ossificans progressiva. It is a highly selective retinoic acid receptor gamma (RARγ) agonist. Daprodustat, a hypoxia-inducible factor prolyl hydroxylase inhibitor, is indicated for the treatment of anemia resulting from chronic kidney disease.

**Figure 18:**
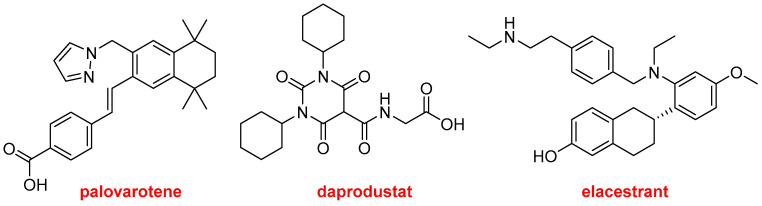
Structures of palovarotene, daprodustat, and elacestrant.

Elacestrant (Orserdu^TM^) (Figure 18) is an estrogen receptor antagonist approved for the treatment of breast cancer.

## 3. Conclusions and Perspectives

COVID-19 has been the main cornerstone of the last three reports of this series. Since the declaration of the COVID-19 pandemic, this is the first report in which the acronym COVID-19 appears tangentially. In 2023, it appears only in regard to Paxlovid^TM^, which contains nirmatrelvir and ritonavir as APIs and was officially approved by the FDA for the treatment of individuals at high risk of progressing to a severe disease status. In 2022, Paxlovid^TM^ received only emergency authorization. An optimistic conclusion regarding COVID-19 is that it will remain with us but like regular flu, without posing a significant high risk for most of the population, who recover from the infection better if vaccinated.

Several drugs approved in previous years, namely the peptides tirzepatide (Mounjaro^TM^ and Zepbound^TM^) and semaglutide (Ozempic^TM^, Rybelsus^TM^, Wegovy^TM^), have been “trending topics” in 2023. While Mounjaro^TM^, Ozempic^TM^, and Rybelsus^TM^ are for the treatment of diabetes, Zepbound^TM^ and Wegovy^TM^ are indicated for fighting the silent pandemic of obesity. Semaglutide has been called the “Hollywood drug” due to its popularity among movie stars. Indeed, widespread popularity has led to a lack of semaglutide for patients with diabetes. Zepbound^TM^ and Wegovy^TM^ cost approximately $US 300 per week, and it is thought that three years of treatment is needed [6]. We are sure that readers will raise moral and ethical questions in relation to this, the answers to which are beyond the purpose of this report.

Related to these two drugs, several new drugs with similar targets are completing clinical phases and are expected to be approved in the coming months. Among them, we can cite retatrutide, which is the first triple agonist of GLP-1, GIP, and GCGR receptors (tirzepatide is a double agonist (GLP-1 and GIP) and semaglutide only a GLP-1 agonist), and mazdutide, pemvidutide, cagrilintide, and survodutide, among others. These kinds of drugs will undoubtedly continue to feature in future reports.

**Figure 19:**
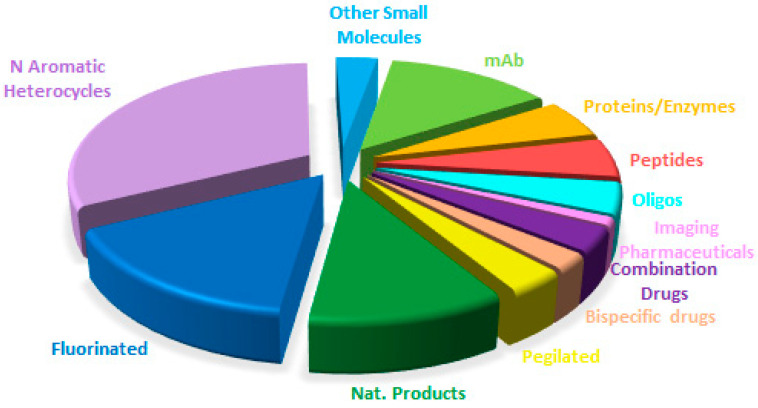
Drugs approved by the FDA in 2023 classified on the basis of chemical structure (drugs can belong to more than one class) Adapted with permission from ref. [1]. Copyright 2022, copyright MDPI.

Figure 19 shows a breakdown of the FDA approvals this year based on the chemical structure of the drugs.

Overall, 2023 has been an extraordinary year in terms of the approval of new drugs (55), surpassed only by 2018, which saw the authorization of 59. The years 2018 and 2023 share the top position in the number of biologics (17) accepted. mAbs, with 12 approvals in 2023, continue again to be the most numerous class of drugs to receive the green light. Two years ago, we gave the honorific title of “2021 Drug of the Year” to aducanumab-avwa (Aduhelm^TM^), which was the first therapy approved by the FDA to directly tackle the biology associated with Alzheimer’s disease. It has not been consolidated on the market due to doubts about its effectiveness. However, this year, the same company, Biogen, in association with Eisai, has launched lecanemab (Leqembi^TM^), which has the same target. No ADC has been added to the list this year, thus stabilizing the number of these drugs available at 14.

Once again, TIDES, with nine new drugs (five peptides and four oligos), continues to be an important class of drug. It is important to highlight nedosiran (Rivfloza^TM^), which contains 58 nucleotides with intrinsic synthetic difficulty. The high number of oligonucleotides approved opens the door for more members of this class of drugs to reach the market in the coming years.

Considering biologics and TIDES together, the total number of 26 drugs accounts for almost 50% of all drugs approved, to the detriment of the so-called small molecules, which a few years ago accounted for more than 80% of all drugs that reached the market.

This year, three pegylated drugs have been approved: the enzyme pegunigalsidase alfa (Elfabrio^TM^); the peptide zilucoplan (Zilbrysq^TM^); and the oligonucleotide avacincaptad pegol (Izervay^TM^). In 2022, only the peptide tirzepatide (Mounjaro^TM^), containing two mini PEGs in its side chain, received authorization. In 2021, five approved drugs were pegylated. This year’s approvals could signify consolidation of the return of pegylated drugs after the withdrawal of peginesatide from the market in 2013.

In 2022, four drugs showed bispecificity, and in 2023, two mAbs [glofitamab-gxbm (Columvi^TM^) and elranatamab (Elrexfio^TM^)] reflect the increasing importance of this kind of drug. Since 2014, nine drugs with bispecificity have been approved. A similar trend is observed for combination drugs, with three approvals in 2023, a similar number to that of the two previous years (three in 2022 and four in 2021).

Natural products, with 10 FDA approvals, continue to be a crucial source of inspiration for drug development. Four of these products belong to the steroid class, which is one of the most frequent on the market. The presence of N-aromatic-heterocycle- and F-based drugs continues to be a constant, and in many cases, both motives are present in the same molecule.

**Figure 20:**
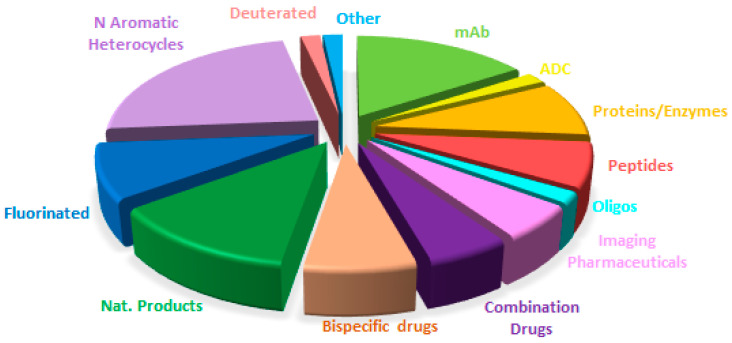
Similar to Figure 19 for 2022, taken with permission from reference [2]. Copyright 2023, copyright MDPI.

**Figure 21:**
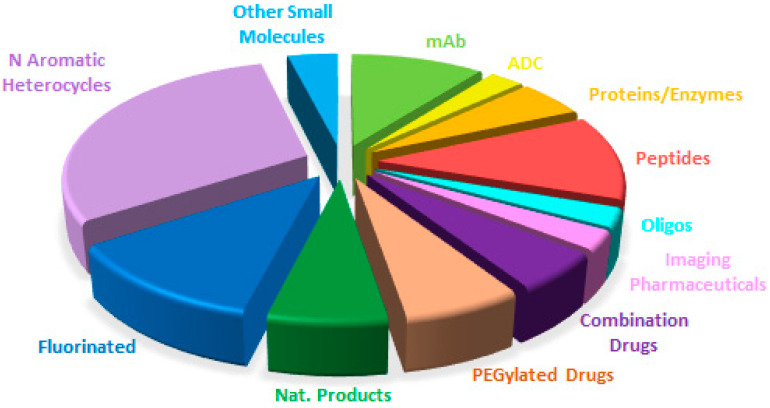
Similar to Figure 19 for 2021, taken with permission from reference [7]. Copyright 2022, MDPI.

Overall, a comparison of the drug authorizations this year (Figure 19) with the last two years (Figure 20 for 2022 and Figure 21 for 2021) reveals a very similar distribution, with a constant growth of biologicals and TIDES to the detriment of small molecules. These observations reflect the well-established trends in a mature sector.

Oncology continues to be the main indication for the drugs approved by the FDA, followed by infectious diseases, rare diseases, and others such as dermatology and sexual-related diseases. Kinase inhibitors are also the most frequent mode of action.

We confer the honorific title of “2023 Drug of the Year” to lecanemab (Leqembi^TM^) due to the great expectations raised for combating a disease that affects large populations around the world.

In conclusion, we envisage an excellent outlook for the pharmaceutical industry in the coming years. The consolidation of mAbs, peptides, and oligonucleotides as APIs widens the scope of new drugs. In coming years, we also foresee the release of the first drugs made by the Chinese pharmaceutical industry onto the market.

On a negative note, we must point out once again that many of these new drugs have prices reaching five or even six digits, which are not affordable for the majority of the population.

## Figures and Tables

**Table 1:** Biologics approved by the FDA in 2023 [1].

Trade Name ^a^	Active Ingredient ^a^	Class	Indication
Beyfortus™	Nirsevimab-alip	mAb	Respiratory syncytial virus (RSV)
Bimzelx™	Mimekizumab	mAb	Moderate to severe plaque psoriasis
Columvi™	Glofitamab-gxbm	mAb	Diffuse large B-cell lymphoma
Elfabrio™	Pegunigalsidase alfa-iwxj	Enzyme	Fabry disease
Elrexfio™	Elranatamab-bcmm	mAb	Relapsed or refractory multiple myeloma
Epkinly™	Epcoritamab-bysp	mAb	Diffuse large and high-grade B-cell lymphoma
Lamzede™	Velmanase alfa-tycv	Enzyme	Non-central nervous system manifestations of alpha-mannosidosis
Leqembi™	Lecanemab	mAb	Alzheimer’s disease
Loqtorzi™	Toripalimab-tpzi	mAb	Recurrent or metastatic nasopharyngeal carcinoma
Ngenla™	Somatrogon-ghla	Glycosylated protein	Growth failure due to inadequate secretion of endogenous growth hormone
Omvoh™	Mirikizumab-mrkz	mAb	Ulcerative colitis
Pombiliti™	Cipaglucosidase alfa-atga	Enzyme	Late-onset Pompe disease
Rystiggo™	Rozanolixizumab-noli	mAb	Myasthenia gravis
Ryzneuta™	Efbemalenograstim alfa-vuxw	Protein	Chemotherapy-induced neutropenia
Talvey™	Talquetamab-tgvs	mAb	Relapsed or refractory multiple myeloma
Veopoz™	Pozelimab-bbfg	mAb	CD55-deficient protein-losing enteropathy (PLE), known as CHAPLE disease
Zynyz™	Retifanlimab-dlwr	mAb	Merkel cell carcinoma

^a^ Trade name used in the USA.

## Data Availability

Not applicable.
